# Appraisal of congenital heart disease guidelines and consensus

**DOI:** 10.3389/fped.2026.1838468

**Published:** 2026-07-24

**Authors:** Maozheng Xuan, Daoxin Wang, Taibing Fan, Weijie Liang, Nan Liu, Haoju Dong, Dong Liang, Menghao Li, Heqi Zhang

**Affiliations:** 1 Department of Children’s Heart Center, Central China Fuwai Hospital of Zhengzhou University, Zhengzhou, China; 2 Medical College of Zhengzhou Sias University, Zhengzhou, Henan, China; 3 China Institute of Environment and Health, South China Hospital, Medical School, Shenzhen University, Shenzhen, China

**Keywords:** congenital heart disease, clinical practice guidelines, expert consensus, AGREE II, RIGHT checklist, quality appraisal, methodological quality, reporting quality

## Abstract

**Objective:**

This is a systematic review of methodological and reporting quality of CHD guidelines and consensus statements.To systematically appraise the methodological and reporting quality of global congenital heart disease (CHD) guidelines and consensus statements, identifying key areas for improvement with implications for international guideline development.

**Methods:**

We searched Chinese and English databases up to November 2025. Four appraisers independently evaluated documents using AGREE II and RIGHT tools. Inter-rater reliability was assessed via ICC; subgroup analyses examined document type, methodology, developer number, and region. The systematic review protocol was registered with PROSPERO (CRD420261415315).

**Results:**

Among 33 included documents (17 CPGs, 16 ECSs), AGREE II scores varied substantially, highest in “Scope and Purpose” (58.42%) and lowest in “Applicability” (32.35%). RIGHT assessment showed sufficient basic reporting but critical gaps in evidence summaries, benefit-harm analyses, and update plans. Guidelines, evidence-based documents, and those with ≥50 developers scored higher. International guidelines outperformed Chinese ones in all quality domains (ICCs > 0.75).

**Conclusion:**

Current CHD guidance documents are purposeful but deficient in rigor, applicability, and transparency. Chinese guidelines lag behind international standards. Future development must adopt evidence-based methods, emphasize applicability, mandate patient and family engagement and improve reporting to effectively translate evidence into practice.

**Systematic Review Registration:**

https://www.crd.york.ac.uk/prospero/display_record.php?ID=CRD420261415315, PROSPERO CRD420261415315.

## Highlights

1. First systematic appraisal of methodological and reporting quality for congenital heart disease guidelines and expert consensus globally.
2. Identified critical deficiencies in applicability, development rigor, and stakeholder involvement despite clear scope and purpose.
3. Chinese guidelines significantly lag behind international standards in methodological and reporting quality.
4. Evidence-based guidelines with ≥50 developers scored higher, underscoring the value of structured methodology and broad collaboration.
5. Provides actionable recommendations for improving future Chinese guideline development using AGREE II and RIGHT frameworks.

## 1 Introduction

Congenital heart disease (CHD) represents one of the most prevalent birth defects globally, exerting a substantial impact on infant survival rates and long-term clinical outcomes (1). Over recent decades, advancements in diagnostic modalities and therapeutic interventions have markedly enhanced the detection rate and treatment efficacy for CHD (2). However, the inherent complexity of the disease spectrum and the diversity of available management strategies impose increasingly sophisticated demands on clinical decision-making.

In response to these challenges, numerous clinical practice guidelines and expert consensus statements (hereafter collectively referred to as guidance documents) addressing various aspects of CHD care—including screening, diagnosis, treatment, and long-term management—have been promulgated by professional societies and academic organizations worldwide. These documents aim to standardize clinical approaches, reduce practice variation, and ultimately improve patient prognosis (3). Nonetheless, significant heterogeneity exists among these publications concerning their developmental methodology, evidentiary foundations, and reporting standards, resulting in variable quality that may undermine their scientific credibility and practical utility (4).

Quality deficiencies in clinical guidance documents are not new. In 2000, Grilli et al. found that 88% of 431 specialty society guidelines lacked literature search strategies and only 5% met three basic quality criteria—an “unsatisfactory” finding (5). Subsequent studies confirm persistence: Kovacevic et al. reported a median AGREE II score of 36% for 74 Croatian national guidelines, with applicability as low as 15%; Jhawar et al. found that >50% of recommendations across 11 of 14 specialties were supported by the lowest evidence level (C); and Nagavci et al. showed that 66% of 98 scientific societies rely on expert opinion—mostly to fill evidence gaps—without standardized definitions (6–8). Thus, despite decades of methodological progress, guideline quality remains a persistent global challenge, particularly for the complex and rapidly evolving field of CHD.

Well-constructed, methodologically rigorous guidelines serve as invaluable tools for clinicians, providing evidence-based frameworks that facilitate standardized care, optimize therapeutic decision-making, enhance treatment outcomes, and improve patients' quality of life. Conversely, recommendations derived from less stringent methodologies or inadequately reported processes may engender clinical inconsistencies, potentially lead to suboptimal care pathways, inefficient allocation of healthcare resources, and adverse impacts on patient outcomes (9). Therefore, ensuring methodological robustness, scientific integrity, and transparent reporting is paramount in the development and dissemination of CHD-related guidance documents.

The WHO Handbook for Guideline Development (2nd edition, 2014) represents the international gold standard. It mandates systematic evidence synthesis, GRADE methodology, rigorous conflict-of-interest management, multidisciplinary stakeholder engagement, and explicit implementation planning. The Guidelines Review Committee (GRC) ensures adherence to these standards, and studies show GRC-approved guidelines score significantly higher in the “rigour of development” domain. Therefore, this study uses the WHO Handbook as the normative benchmark to assess current CHD guideline quality (9).

The Appraisal of Guidelines for Research and Evaluation (AGREE) instrument, particularly its refined version AGREE II, has emerged as the international standard for the systematic assessment of guideline methodological quality (10). Concurrently, the Reporting Items for practice Guidelines in HealThcare (RIGHT) checklist provides a comprehensive framework for evaluating the transparency and completeness of guideline reporting (11). This study employs both the AGREE II instrument and RIGHT checklist to conduct a systematic evaluation of the methodological and reporting quality of published CHD-related clinical guidelines and consensus statements. The primary objectives are to identify prevailing strengths and weaknesses in current guidance documents, and to furnish evidence-based recommendations for the future development and revision of high-quality, clinically applicable CHD guidelines in China.

## 2 Materials and methods

### 2.1 Inclusion and exclusion criteria

**Inclusion Criteria:**

1. Clinical practice guidelines or expert consensus statements publicly available via academic journals, official medical organization websites, or professional conference platforms, with a primary focus on “congenital heart disease” or “congenital heart defects.”
2. Documents whose core content addresses at least one aspect of CHD management: screening, diagnosis, assessment, medical therapy, interventional therapy, surgical intervention, perioperative management, long-term follow-up, or comprehensive care.
3. For documents with explicit updated versions issued by the same organization, only the most recent version was included.

**Exclusion Criteria:**

1. Documents not published in Chinese or English.
2. Interpretations, translations, summaries, or commentaries on existing guidelines/consensus statements.
3. Documents for which full text or essential information (e.g., development methods, recommendations) was inaccessible or incomplete.
4. Indirect publications such as guideline introductions, methodological critiques, implementation analyses, application guidance, news reports, or errata notices.

### 2.2 Search strategy

A comprehensive systematic search was conducted across Chinese and English databases and specialized guideline platforms to identify all relevant CHD clinical practice guidelines and consensus statements.The complete search strategies for each database are provided in the PROSPERO protocol (CRD420261415315) and are also available as Supplementary File S1. The following resources were searched from their inception to November 2025:

**English Databases:** PubMed, Embase, Web of Science

**Chinese Databases:** China Biology Medicine disc (CBM), WanFang Data, China National Knowledge Infrastructure (CNKI)

**Guideline Platforms:** Guidelines International Network (GIN), National Institute for Health and Care Excellence (NICE) website, American Heart Association (AHA) and American College of Cardiology (ACC) official websites, and the Yimaitong Guideline Channel.

Search terms combined Medical Subject Headings (MeSH) and free-text keywords. The following are representative examples:

**Chinese Search Terms:** 先天性心脏病, 先天性心脏缺陷, 心脏畸形, 指南, 共识, 实践指南, 专家共识, 推荐意见, 管理规范

**English Search Terms:** “Congenital Heart Disease,” “Congenital Heart Defect,” “Heart Defect, Congenital,” “guideline,” “consensus,” “practice guideline,” “expert consensus,” “recommendation,” “statement”.

### 2.3 Methodological reference standard

The WHO Handbook (2nd edition) served as our methodological standard. Its 10-chapter framework spans planning, PICO questions, systematic reviews, GRADE, balanced recommendations, COI management, GRC peer review, dissemination, and transparency (9). We used AGREE II and RIGHT to assess all documents against these WHO-endorsed criteria, which cover evidence synthesis, stakeholder engagement, applicability, and reporting.

### 2.4 Study selection and data extraction

Two researchers independently screened titles, abstracts, and subsequently full texts according to the predefined criteria. Discrepancies were resolved through discussion or consultation with a third researcher. For missing information, efforts were made to contact the corresponding authors. GRADE methodology, mandated by the WHO Handbook, served as the reference standard for assessing evidence quality and recommendation strength in included documents; consistent GRADE adoption indicates adherence to WHO standards (9).

**Data extraction included:**

1. Basic document information: title, publication journal/year, issuing organization, development methodology.
2. Key elements for methodological quality assessment.
3. Key elements for reporting quality assessment.

### 2.5 Quality assessment

Four trained researchers with doctoral-level expertise independently evaluated the included documents using the AGREE II instrument and RIGHT checklist. AGREE II comprises 23 items across six domains. Each item is scored on a 7-point Likert scale (1 = strongly disagree to 7 = strongly agree). Domain scores were calculated as standardized percentages: (Obtained Score − Minimum Possible Score)/(Maximum Possible Score − Minimum Possible Score) × 100%.

The RIGHT checklist includes 22 items across seven domains. Each item is rated as “reported” (1 point), “partially reported” (0.5 points), or “not reported” (0 points). Total scores were calculated accordingly.

### 2.6 Statistical analysis

Descriptive analyses were performed using Excel. SPSS 26.0 was employed for statistical testing. The intraclass correlation coefficient (ICC) was calculated to assess inter-rater reliability for AGREE II scores. ICC values were interpreted as follows: <0.40 = poor reliability; 0.40–0.75 = moderate reliability; >0.75 = excellent reliability. Between-group comparisons were analyzed using non-parametric tests (Mann–Whitney *U* test). A two-tailed *P*-value <0.05 was considered statistically significant.

### 2.7 Protocol registration

This systematic review was registered with PROSPERO (International Prospective Register of Systematic Reviews; registration number: CRD420261415315). The registered protocol detailed the research question, search strategy, inclusion and exclusion criteria, data extraction procedures, quality appraisal methods (AGREE II and RIGHT), and planned subgroup analyses.

## 3 Results

### 3.1 Study selection

**Figure 1:**
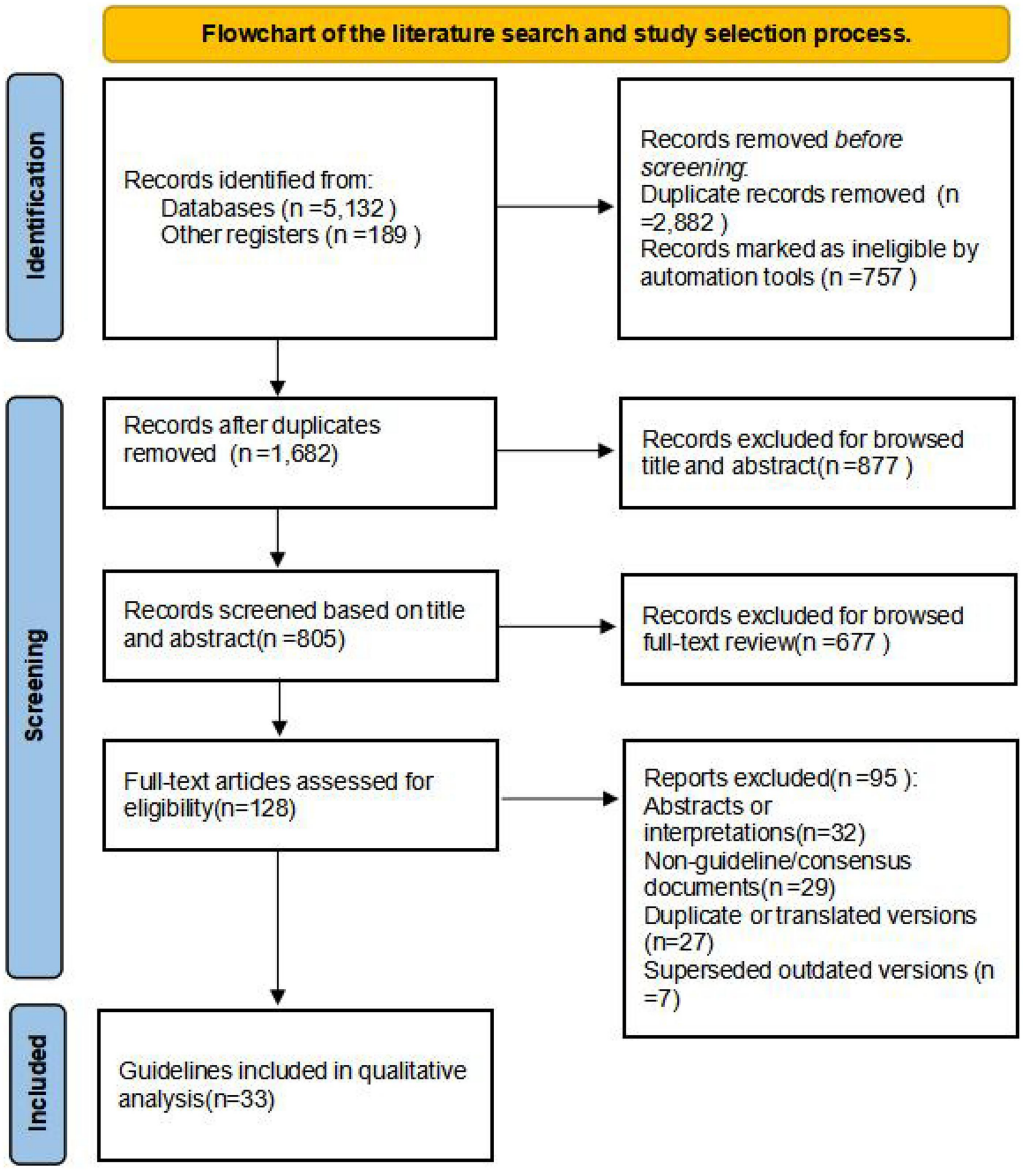
Flowchart of the literature search and study selection process.

The initial search yielded 5, 326 citations. After deduplication and sequential screening of titles/abstracts and full texts, 33 documents (clinical guidelines and consensus statements) met the inclusion criteria (1–4, 11–39). No full-text articles were excluded; all records that did not meet the inclusion criteria were excluded during title/abstract screening. The study selection process is summarized in Figure 1.

**Figure 2:**
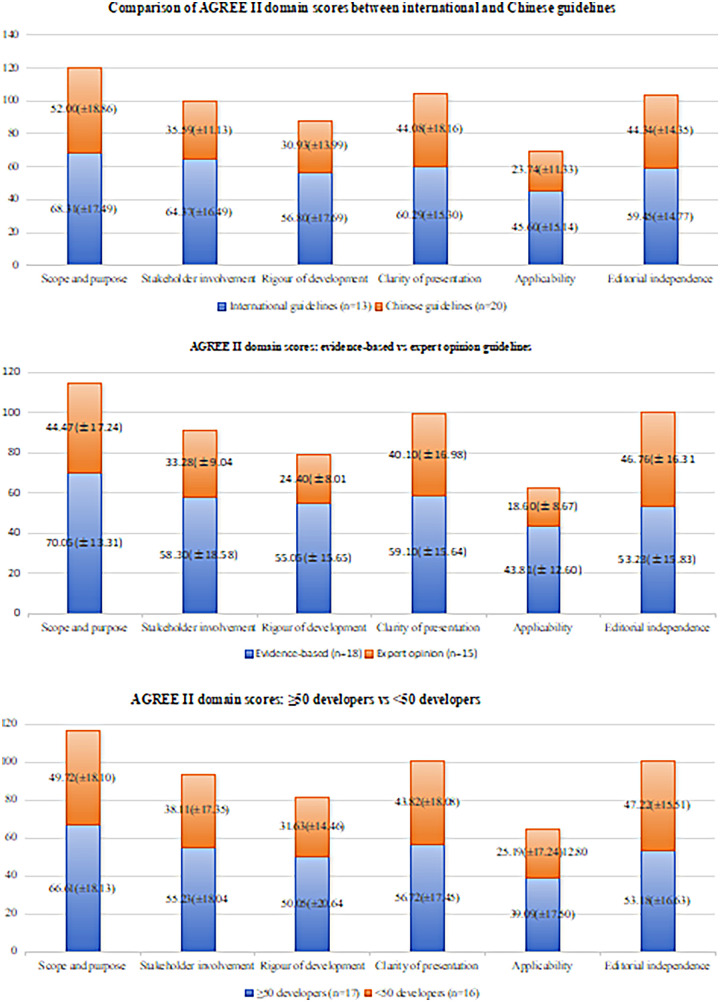
Subgroup comparisons of AGREE II domain scores across document characteristics. Comparison of AGREE II domain scores by subgroup: international vs. Chinese. evidence-based vs. expert opinion, and ≥50 vs. <50 developers.

### 3.2 Characteristics of included articles

Among the 33 included documents, 17 (51.51%) were clinical guidelines and 16 (48.48%) were consensus statements. 17 documents (51.51%) involved ≥50 developers, while 16 (48.48%) involved <50. 18 documents (54.54%) were developed using evidence-based methods, whereas 15 (45.45%) relied primarily on expert opinion or narrative review. 20 documents (60.60%) originated from China, and 13 (39.39%) were international (predominantly European/American). 31 documents (93.93%) were issued by professional societies/associations, and two (6.06%) were published by individual authors. The detailed characteristics are presented in Table 1.

**Table 1:** Basic characteristics of the included documents.

No	Guideline/consensus title	Year	Type	Issuing organization	Developers (n)	References (n)	Pages	Development method
1	2020 ESC Guidelines for the management of adult congenital heart disease (1)	2020	Guideline	European Society of Cardiology	145	329	83	Evidence-based
2	2017 ESC/EACTS Guidelines for the Management of Valvular Heart Disease (2)	2017	Guideline	ESC/European Association for Cardio-Thoracic Surgery	72	211	53	Evidence-based
3	2018 ESC Guidelines for the management of cardiovascular diseases during pregnancy (3)	2018	Guideline	ESC/European Society of Anaesthesiology/European Society of Gynaecology	77	-	82	Evidence-based
4	2018 AHA/ACC Guideline for the Management of Adults With Congenital Heart Disease (4)	2019	Guideline	American College of Cardiology/American Heart Association	61	526	61	Evidence-based
5	2022 ACC/AHA Guideline for the Diagnosis and Management of Aortic Disease (11)	2022	Guideline	AHA/ACC	81	1,548	149	Evidence-based
6	2023 ESC Guidelines for the management of cardiomyopathies (12)	2023	Guideline	European Society of Cardiology	162	1,204	124	Evidence-based
7	2022 ESC/ERS Guidelines for the diagnosis and treatment of pulmonary hypertension (13)	2022	Guideline	ESC/European Respiratory Society	165	855	114	Evidence-based
8	The AATS 2022 Expert Consensus Document: Management of infants and neonates with tetralogy of Fallot (14)	2022	Consensus	American Association for Thoracic Surgery	30	138	30	Expert opinion
9	2016 ESC Guidelines for the management of atrial fibrillation developed in collaboration with EACTS. (15)	2016	Guideline	ESC Atrial Fibrillation Task Force	73	1,042	90	Evidence-based
10	The AATS consensus guidelines on bicuspid aortic valve-related aortopathy (16)	2018	Guideline	American Association for Thoracic Surgery	20	259	34	Evidence-based
11	Cardiac Surgery in Trisomy 13 and 18: A Guide to Clinical Decision-Making (17)	2020	Guideline	Individual	5	47	15	Evidence-based
12	2013 ESH/ESC guidelines for the management of arterial hypertension (18)	2013	Guideline	European Society of Hypertension/ESC	131	735	72	Evidence-based
13	2015 ESC Guidelines for the management of patients with ventricular arrhythmias and the prevention of sudden cardiac death (19)	2015	Guideline	ESC	73	809	87	Evidence-based
14	Chinese Guidelines for the Diagnosis and Treatment of Pulmonary Hypertension (2021) (20)	2021	Guideline	Chinese Thoracic Society	147	268	41	Evidence-based
15	Guidelines for Interventional Therapy of Common Congenital Heart Diseases in Children (2025) (21)	2025	Guideline	Chinese Pediatric Society	94	114	16	Evidence-based
16	Chinese Guidelines for Comprehensive Management of Cardiomyopathy 2025 (22)	2025	Guideline	National Center for Cardiovascular Diseases	75	288	43	Evidence-based
17	Guidelines for Diagnosis and Treatment of Pediatric Congenital Heart Disease-Associated Pulmonary Hypertension (23)	2022	Guideline	Chinese Pediatric Surgery Society	23	74	15	Evidence-based
18	Guidelines for Percutaneous Interventional Therapy of Common Congenital Heart Diseases (2021) (24)	2021	Guideline	National Center for Structural Heart Disease Intervention Quality Control	48	202	23	Evidence-based
19	Chinese Expert Consensus on Ventricular Arrhythmias (2020 updated version) (25)	2020	Consensus	Chinese Society of Pacing and Electrophysiology	87	410	65	Expert opinion
20	Chinese Expert Consensus on Rehabilitation for Children and Adolescents with Congenital Heart Disease (26)	2024	Consensus	National Center for Cardiovascular Diseases	62	83	14	Expert opinion
21	Chinese Expert Consensus on Surgical Treatment of Aortic Valve Disease in Children (27)	2024	Consensus	National Committee of Experts on Congenital Heart Disease	52	47	7	Expert opinion
22	Chinese Expert Consensus on Diagnosis and Treatment of Congenital Heart Disease-Associated Pulmonary Arterial Hypertension (2015) (28)	2015	Consensus	Chinese College of Cardiovascular Physicians	36	100	9	Expert opinion
23	Chinese Expert Consensus on Diagnosis and Treatment of Total Anomalous Pulmonary Venous Connection (29)	2024	Consensus	National Committee of Experts on Congenital Heart Disease	49	53	7	Expert opinion
24	Chinese Expert Consensus on the "Integrated" Prenatal and Postnatal Diagnosis and Treatment Model for Congenital Heart Disease (30)	2022	Consensus	Expert Group on Integrated CHD Diagnosis and Treatment	46	63	7	Expert opinion
25	Expert Consensus on Normal and Abnormal Features of Fetal Cardiac Ultrasound Cross-Sections (31)	2023	Consensus	Chinese Society of Ultrasound in Medicine	42	33	8	Expert opinion
26	Expert Consensus on Perinatal Nursing Management for Children with Congenital Heart Disease (32)	2025	Consensus	National Center for Cardiovascular Diseases	31	42	10	Evidence-based
27	Expert Consensus on Echocardiographic Diagnostic Criteria for New Anatomical Classification of Congenital Double Outlet Right Ventricle (33)	2022	Consensus	Chinese Society of Ultrasound in Medicine	17	23	9	Expert opinion
28	Chinese Expert Clinical Management Consensus for Peri-Anesthetic Care of Cardiac Patients Undergoing Non-Cardiac Surgery (34)	2021	Consensus	Chinese Society of Cardio-Thoracic Anesthesia	32	31	15	Expert opinion
29	Chinese Expert Consensus on Diagnosis of Hypertrophic Cardiomyopathy in Children (35)	2019	Consensus	Chinese Pediatric Society	58	19	6	Expert opinion
30	Chinese Expert Consensus on Cardiovascular Implantable Electronic Devices in Children (36)	2023	Consensus	Chinese Society of Biomedical Engineering	25	84	11	Expert opinion
31	Chinese Expert Consensus on Interventional Therapy for Common Congenital Heart Diseases (37)	2011	Consensus	Chinese College of Cardiovascular Physicians	25	55	33	Expert opinion
32	Chinese Expert Consensus on Surgical Treatment of Congenital Heart Disease (38)	2020	Consensus	National Committee of Experts on Congenital Heart Disease	40	643	85	Expert opinion
33	Chinese Expert Consensus on Surgical Treatment of Congenital Heart Disease: Unilateral Pulmonary Artery Absence (39)	2025	Consensus	Individual	24	17	4	Expert opinion

ESC, European Society of Cardiology; EACTS, European Association for Cardio-Thoracic Surgery; AHA, American Heart Association; ACC, American College of Cardiology; AATS, American Association for Thoracic Surgery; ESH, European Society of Hypertension; ERS, European Respiratory Society.

### 3.3 Methodological quality assessment (AGREE II)

The ICC values for all documents exceeded 0.75, indicating excellent inter-rater agreement (Table 2).

**Table 2:** ICC for AGREE II assessments.

Document No.	ICC (95% CI)	*F*-value	*P*-value
1 (1)	0.756 (0.539–0.886)	4.098	0.000
2 (2)	0.962 (0.928–0.982)	26.363	0.000
3 (3)	0.938 (0.883–0.971)	16.106	0.000
4 (4)	0.973 (0.949–0.987)	37.161	0.000
5 (11)	0.879 (0.772–0.943)	8.294	0.000
6 (12)	0.870 (0.754–0.939)	7.663	0.000
7 (13)	0.915 (0.839–0.960)	11.755	0.000
8 (14)	0.929 (0.866–0.967)	14.054	0.000
9 (15)	0.944 (0.894–0.974)	17.785	0.000
10 (16)	0.902 (0.816–0.954)	10.241	0.000
11 (17)	0.871 (0.738–0.942)	7.761	0.000
12 (18)	0.865 (0.731–0.939)	7.704	0.000
13 (19)	0.903 (0.806–0.956)	10.325	0.000
14 (20)	0.943 (0.886–0.974)	17.522	0.000
15 (21)	0.912 (0.833–0.959)	11.317	0.000
16 (22)	0.947 (0.901–0.975)	18.983	0.000
17 (23)	0.895 (0.802–0.951)	9.532	0.000
18 (24)	0.969 (0.942–0.986)	32.540	0.000
19 (25)	0.936 (0.880–0.970)	15.690	0.000
20 (26)	0.954 (0.913–0.978)	21.667	0.000
21 (27)	0.949 (0.904–0.976)	19.712	0.000
22 (28)	0.961 (0.926–0.982)	25.853	0.000
23 (29)	0.972 (0.947–0.987)	35.410	0.000
24 (30)	0.934 (0.876–0.969)	15.243	0.000
25 (31)	0.920 (0.850–0.963)	12.564	0.000
26 (32)	0.881 (0.776–0.944)	8.420	0.000
27 (33)	0.918 (0.846–0.962)	12.256	0.000
28 (34)	0.933 (0.873–0.969)	14.909	0.000
29 (35)	0.905 (0.818–0.956)	10.510	0.000
30 (36)	0.864 (0.743–0.936)	7.360	0.000
31 (37)	0.909 (0.826–0.958)	11.021	0.000
32 (38)	0.949 (0.903–0.976)	19.475	0.000
33 (39)	0.971 (0.945–0.986)	34.340	0.000

ICC, Intraclass Correlation Coefficient; CI, Confidence Interval.

#### 3.3.1 Overall AGREE II results

The methodological quality across the six AGREE II domains was imbalanced (Table 3):

- **Scope and purpose:** Highest scoring domain (average 58.42%). 23 documents scored >50%. Most clearly described overall objectives, specific clinical questions, and target populations.
- **Stakeholder involvement:** Average score was 46.93%. 10 documents scored >50%. 78.79% (26/33) did not explicitly consider patient/public perspectives; 84.85% (28/33) inadequately described panel composition and roles.
- **Rigour of development:** Core methodological domain with the second-lowest average (41.12%). Only 10 documents scored >50%. Major deficiencies: 72.73% lacked clear evidence inclusion/exclusion criteria; 81.82% did not critically appraise evidence limitations; 75.76% failed to systematically assess recommendation benefits/harms.
- **Clarity of Presentation:** Average 50.47%. 16 documents scored >50%. Recommendations were generally clear, but 51.52% lacked tailored recommendations for different clinical scenarios or patient subgroups.
- **Applicability:** Lowest scoring domain (average 32.35%). Only 5 documents scored >50%. Over 60% failed to discuss implementation barriers/facilitators or resource implications; only 24.24% provided implementation tools or evaluation criteria.
- **Editorial Independence:** Average 50.29%. 16 documents scored >50%. Most declared no funding influence, but 63.64% had insufficiently detailed conflict of interest declarations.

**Table 3:** AGREE II domain scores for included documents (%).

No.	Scope & purpose	Stakeholder involvement	Rigour of development	Clarity	Applicability	Editorial Independence	Average	Recommendation
1 (1)	86.11	70.83	76.56	55.55	64.28	83.33	72.77	A
2 (2)	43.05	84.81	62.50	49.36	35.41	60.41	55.92	B
3 (3)	63.88	31.94	60.41	54.16	38.54	75.00	53.98	A
4 (4)	64.55	62.50	85.41	86.11	56.25	31.25	64.34	A
5 (11)	72.22	51.38	67.70	79.16	65.62	45.83	63.65	A
6 (12)	88.88	79.16	79.16	52.77	49.10	60.41	68.24	A
7 (13)	90.27	73.61	48.95	54.16	60.71	77.08	67.46	A
8 (14)	34.72	47.22	32.29	56.96	10.41	66.66	41.37	B
9 (15)	61.11	65.27	40.10	66.66	43.75	58.33	55.87	A
10 (16)	54.16	64.55	30.72	41.66	40.62	39.58	45.21	B
11 (17)	83.33	86.11	62.50	84.81	54.16	56.25	71.19	A
12 (18)	80.55	75.00	45.31	64.55	38.54	64.58	61.42	A
13 (19)	65.27	44.44	46.87	37.97	35.41	54.16	47.35	B
14 (20)	75.00	36.11	47.39	67.08	46.87	47.91	53.39	B
15 (21)	75.00	54.16	52.08	79.16	34.37	52.08	57.80	A
16 (22)	72.22	59.72	50.00	63.29	37.50	50.00	55.45	A
17 (23)	48.61	22.22	52.08	38.88	33.33	43.75	39.81	B
18 (24)	72.22	47.22	56.25	43.03	37.50	25.00	46.87	B
19 (25)	61.11	43.05	25.52	36.70	23.95	20.83	35.19	B
20 (26)	54.16	44.44	18.22	61.11	15.62	45.83	39.89	B
21 (27)	63.88	40.27	31.25	38.88	12.50	45.83	38.76	B
22 (28)	50.00	36.11	10.93	34.72	32.29	56.25	36.71	B
23 (29)	68.05	31.64	17.70	44.30	12.50	33.33	34.58	B
24 (30)	55.55	31.94	31.25	44.44	21.87	51.46	39.41	B
25 (31)	56.94	39.24	26.56	46.83	35.41	75.00	46.66	B
26 (32)	64.55	40.50	27.08	45.56	16.66	33.33	37.94	B
27 (33)	55.55	36.11	33.85	54.16	23.95	27.08	38.45	B
28 (34)	48.61	31.94	27.08	70.88	21.87	31.25	38.60	B
29 (35)	15.27	22.22	13.54	17.72	6.25	31.25	17.70	C
30 (36)	31.94	26.38	28.12	31.64	10.41	47.91	29.40	C
31 (37)	20.83	20.25	17.18	20.25	19.79	62.50	26.80	B
32 (38)	27.77	16.45	35.93	35.44	8.33	39.58	27.25	C
33 (39)	22.78	31.94	16.66	7.59	23.95	66.66	28.26	B
Average	58.42	46.93	41.12	50.47	32.35	50.29	46.59	—

Recommendation categories based on AGREE II: A (strongly recommended), B (recommended with modifications), C (not recommended).

#### 3.3.2 Subgroup analysis (AGREE II)

Subgroup comparisons revealed significant patterns (Table 4):

- Guidelines vs. Consensus: Guidelines scored significantly higher than consensus statements across all domains except Editorial Independence (*P* < 0.05).
- Developer Numbers: Documents with ≥50 developers scored significantly higher than those with <50 across five domains (*P* < 0.05).
- Development Methodology: Evidence-based documents significantly outperformed expert opinion-based documents across five domains (*P* < 0.05).
- Geographic Origin: International (European/American) documents significantly outperformed Chinese documents across all six domains (*P* < 0.05).
- Issuing Organization: No significant difference between society/association vs. individually authored documents (*P* > 0.05), likely due to small sample size of individual documents.

**Table 4:** Subgroup analysis of AGREE II domain score.

Subgroup		Scope & Purpose	Stakeholder Involvement	Rigour of Development	Clarity	Applicability	Editorial Independence
Guidelines (*n* = 17)	Score Range	43.05–90.27	22.22–86.11	30.72–85.41	37.97–86.11	33.33–65.62	25.00–83.33
	Mean ± SD	70.37 ± 13.65	59.35 ± 18.60	56.70 ± 14.44	59.90 ± 15.74	45.40 ± 10.95	54.40 ± 15.50
Consensus (*n* = 16)	Score Range	15.27–68.05	16.45–47.22	10.93–35.93	7.59–70.88	6.25–35.41	20.83–75.00
	Mean ± SD	45.73 ± 17.40	33.73 ± 8.92	24.57 ± 7.77	40.44 ± 16.46	18.48 ± 8.39	45.92 ± 16.11
*P*-value		0.000	0.000	0.000	0.002	0.000	0.133
≥50 Developers (*n* = 17)	Score Range	15.27–90.27	22.22–84.81	13.54–85.41	17.72–86.11	6.25–65.62	20.83–83.33
	Mean ± SD	66.61 ± 18.13	55.23 ± 18.04	50.05 ± 20.64	56.72 ± 17.45	39.09 ± 17.50	53.18 ± 16.63
<49 Developers (*n* = 16)	Score Range	20.83–83.33	16.45–86.11	10.93–62.50	7.59–84.81	8.33–54.16	25.00–75.00
	Mean ± SD	49.72 ± 18.10	38.11 ± 17.35	31.63 ± 14.46	43.82 ± 18.08	25.19 ± 12.80	47.22 ± 15.51
*P*-value		0.012	0.009	0.006	0.045	0.014	0.296
Evidence-based (*n* = 18)	Score Range	43.05–90.27	22.22–86.11	27.08–85.41	37.97–86.11	16.66–65.62	25.00–83.33
	Mean ± SD	70.05 ± 13.31	58.30 ± 18.58	55.05 ± 15.65	59.10 ± 15.64	43.81 ± 12.60	53.23 ± 15.83

Subgroup	Scope & purpose	Stakeholder involvement	Rigour of development	Clarity	Applicability	Editorial independence	Subgroup
Expert opinion (*n* = 15)	Score Range	15.27–68.05	16.45–47.22	10.93–35.93	7.59–70.88	6.25–35.41	20.83–75.00
	Mean ± SD	44.47 ± 17.24	33.28 ± 9.04	24.40 ± 8.01	40.10 ± 16.98	18.60 ± 8.67	46.76 ± 16.31
*P*-value		0.000	0.000	0.000	0.002	0.000	0.257
International (*n* = 13)	Score Range	34.72–90.27	31.94–86.11	30.72–85.41	37.97–86.11	10.41–65.62	31.25–83.33
	Mean ± SD	68.31 ± 17.49	64.37 ± 16.49	56.80 ± 17.69	60.29 ± 15.30	45.60 ± 15.14	59.45 ± 14.77
Chinese (*n* = 20)	Score Range	15.27–75.00	16.45–59.72	10.93–56.25	7.59–79.16	6.25–46.87	20.83–75.00
	Mean ± SD	52.00 ± 18.86	35.59 ± 11.13	30.93 ± 13.99	44.08 ± 18.16	23.74 ± 11.33	44.34 ± 14.35
*P*-value		0.018	0.000	0.000	0.012	0.000	0.006
society/association (*n* = 31)	Score Range	15.27–90.27	16.45–84.81	10.93–85.41	17.72–86.11	6.25–65.62	20.83–83.33
	Mean ± SD	58.77 ± 18.82	46.15 ± 18.58	41.22 ± 19.74	50.74 ± 16.46	31.92 ± 16.73	49.57 ± 16.34
individually authored (*n* = 2)	Score Range	22.78–83.33	31.94–86.11	16.66–62.50	7.59–84.81	23.95–54.16	56.25–66.66
	Mean ± SD	53.05 ± 42.81	59.02 ± 38.30	39.58 ± 32.41	46.20 ± 54.60	39.05 ± 21.36	61.45 ± 7.36
*P*-value		0.699	0.373	0.912	0.925	0.567	0.321

### 3.4 Reporting quality assessment (RIGHT)

#### 3.4.1 Overall RIGHT results

Reporting quality was highest for basic information items (Items 1a, 2, 4: 100% reported) and lowest for evidence summary tables (Item 14b: 93.93% not reported), analysis of benefits/harms (Item 8b: 81.81% not reported), and update procedures (Item 20: 69.69% not reported). Table 5 summarizes reporting rates.

**Table 5:** RIGHT checklist reporting rate.

Item	Fully reported (%)		Partially reported (%)		Not reported (%)	
	Number (n)	Percentage (%)	Number (n)	Percentage (%)	Number (n)	Percentage (%)
1a. Title	33	100.00	0	0.00	0	0.00
1b. Registration	16	48.48	0	0.00	17	51.51
1c. Authors/contributors	31	93.93	2	6.06	0	0.00
2. Abstract	33	100.00	0	0.00	0	0.00
3. Executive summary	32	96.96	1	3.03	0	0.00
4. Abbreviations	33	100.00	0	0.00	0	0.00
5. Background/rationale	10	30.30	21	63.63	2	6.06
6. Objectives	6	18.18	25	75.75	2	6.06
7a. Guideline users	21	63.63	12	36.36	0	0.00
7b. Target population	4	12.12	7	21.21	22	66.66
8a. Evidence collection	2	6.06	2	6.06	29	87.87
8b. Evidence selection	0	0.00	6	18.18	27	81.81
9a. Recommendation development	8	24.24	3	9.09	22	66.66
9b. Recommendation formulation	9	27.27	9	27.27	15	45.45
10a. Evidence summary	0	0.00	8	24.24	25	75.75
10b. Evidence quality	0	0.00	5	15.15	28	84.84
11a. Recommendations	21	63.63	10	30.30	2	6.06
11b. Recommendation strength	7	21.21	13	39.39	13	39.39
12. Rationale for recommendations	9	27.27	18	54.54	6	18.18
13a. Implementation considerations	7	21.21	23	69.69	3	9.09
13b. Implementation tools	0	0.00	10	30.30	23	69.69
13c. Monitoring/auditing	28	84.84	2	6.06	3	9.09
14a. Benefits/harms	20	60.60	6	18.18	7	21.21
14b. Evidence summary table	0	0.00	2	6.06	31	93.93
14c. Resource implications	0	0.00	6	18.18	27	81.81
15. Recommendations clarity	24	72.72	6	18.18	3	9.09
16. External review	7	21.21	9	27.27	17	51.51
17. Updating procedure	8	24.24	11	33.33	14	42.42
18a. Funding sources	20	60.60	2	6.06	11	33.33
18b. Funders’ role	0	0.00	8	24.24	25	75.75
19a. COI declaration	29	87.87	4	12.12	0	0.00
19b. COI management	14	42.42	3	9.09	16	48.48
20. Guideline status/updates	8	24.24	2	6.06	23	69.69
21. Access/contact	9	27.27	5	15.15	19	57.57
22. Other information	11	33.33	6	18.18	16	48.48

#### 3.4.2 Subgroup analysis (RIGHT)

RIGHT total scores followed similar subgroup patterns (Table 6):

- **Guidelines** (11.30 ± 1.84) significantly outperformed consensus (6.60 ± 0.89) (*P* < 0.001).
- Documents with **≥50 developers** (10.76 ± 2.43) scored higher than those with <50 developers (7.46 ± 2.08) (*P* < 0.001).
- **Evidence-based documents** (11.27 ± 1.89) scored higher than expert opinion documents (6.63 ± 0.93) (*P* < 0.001).
- **International documents** (11.96 ± 1.74) scored higher than Chinese documents (7.35 ± 1.54) (*P* < 0.001).

**Table 6:** Subgroup analysis of RIGHT total scores.

Subgroup	Score Range	Mean ± SD	*P*-value
Guidelines/Consensus			
Guidelines (*n* = 17)	9.00–25.50	17.79 ± 5.78	0.000
Consensus (*n* = 16)	3.00–11.00	6.37 ± 2.83	
number of Developers			
≥50 Developers (*n* = 17)	6.00–25.50	17.08 ± 6.56	0.000
<50 Developers (*n* = 16)	3.00–15.00	7.12 ± 3.85	
Formulate the method			
Evidence-based (*n* = 18)	4.00–25.50	17.02 ± 6.48	0.000
Expert opinion (*n* = 15)	3.00–11.00	6.53 ± 2.85	
Formulate the country			
International (*n* = 13)	9.50–25.50	19.38 ± 5.70	0.000
Chinese (*n* = 20)	3.00–13.50	7.62 ± 3.63	
Affiliation			
society/association (*n* = 31)	3.00–25.50	12.54 ± 7.41	0.380
individually authored (*n* = 2)	3.50–12.00	7.75 ± 6.01	

## 4 Discussion

This comprehensive evaluation of 33 CHD-related clinical guidelines and consensus statements using AGREE II and RIGHT instruments reveals significant quality variations, with pronounced deficiencies in core methodological and reporting domains. While “Scope and Purpose” was relatively well-addressed, critical weaknesses were identified in “Rigour of Development,” “Applicability,” and “Stakeholder Involvement”—findings consistent with quality appraisals in other medical fields (40). However, the CHD domain presents unique challenges due to disease heterogeneity, rapid technological evolution, and the need for lifelong patient management, which may exacerbate these methodological shortcomings.

Applying WHO Handbook standards reveals that while Scope and Purpose (58.42%) approximates international acceptability, Rigour of Development (41.12%) falls far below WHO requirements. This gap is concerning: Burda et al. found that WHO GRC-approved guidelines scored significantly higher in rigour of development, suggesting that better adherence to the Handbook and GRC oversight could improve quality (9).

The subgroup analyses provide actionable insights. The superior performance of guidelines over consensus statements underscores the importance of formal guideline development frameworks. The marked advantage of evidence-based methodologies highlights the necessity of systematic evidence synthesis rather than reliance on expert opinion alone. The positive association between developer numbers and quality suggests that multidisciplinary collaboration enhances comprehensiveness. Most notably, the substantial gap between international and Chinese documents indicates systematic deficiencies in China's guideline development ecosystem.

Our findings align with a recent systematic review by Gomes et al., who assessed the rigour of development of ESC and ACC/AHA guidelines from 2013 to 2024 using the AGREE II third domain (41). They reported a mean score of 29 out of 56, indicating substantial methodological deficiencies, and noted that ACC/AHA guidelines scored significantly higher than ESC guidelines (36 vs. 24). In our study, among the 13 international guidelines (which include ESC and ACC/AHA documents), we observed a comparable pattern: the AGREE II third domain scores for ACC/AHA guidelines were consistently higher than those for ESC guidelines. Moreover, our study extends these findings in several important ways. First, we evaluated all six AGREE II domains, revealing that “Applicability” and “Rigour of development” were the poorest-performing domains globally. Second, we included expert consensus statements and demonstrated that their methodological quality is substantially lower than that of formal guidelines. Third, we assessed reporting quality using the RIGHT checklist, identifying critical gaps in evidence summaries, benefit-harm analyses, and update procedures. Fourth, we included Chinese guidelines and found a significant quality gap compared with international standards, highlighting an urgent need for capacity building in China. Together, our study and that of Gomes et al. underscore that even the most influential cardiovascular guidelines have substantial room for improvement in methodological rigour and transparency, and that this issue is even more pronounced in China and in consensus-type documents.Due to the limited number of ACC/AHA guidelines in our sample (*n* = 2, out of 13 international guidelines) and the scattered publication years, we did not generate a separate figure for the year-by-year trend. Instead, we present the comparison in text, noting that our observed pattern (ACC/AHA > ESC) is fully consistent with Gomes et al.Importantly, our subgroup analysis only compared Chinese guidelines with European/North American ones. However, guidelines from other emerging economies—such as India's first national CHD guidelines (2019–2020)—remain unexplored (42). Future research should systematically appraise such regions, as their experiences in balancing evidence with resource constraints may offer more relevant lessons for China than those derived solely from high-resource settings. Three major areas require urgent attention. (1) Strengthenthe methodological rigour, particularly regarding transparent evidence selection criteria, critical appraisal of evidence limitations, and explicit linkage between recommendations and their evidentiary foundations (43). (2) Enhance the practical applicability; guidelines should proactively address implementation barriers, resource implications, and provide tools for clinical integration (44). (3) Improve the reporting transparency, especially concerning evidence summary tables, benefit-harm analyses, and update procedures.

Based on these findings, we propose four strategic recommendations for improving CHD guideline quality in China:

1. Adopt International Standard Methodologies: Chinese guideline developers should fully implement structured evidence-based processes as outlined by AGREE II and GRADE (Grading of Recommendations Assessment, Development and Evaluation) frameworks (45).
2. Enhance Practical Implementation Design: Guidelines should be developed with explicit “implementation plans” addressing local contextual factors, resource availability, and monitoring indicators (46).
3. Establish Dynamic Update Mechanisms: Given rapid advancements in CHD management, formal processes for periodic review and updating (e.g., every 3–5 years) should be institutionalized.
4. Strengthen Capacity Building: Investment in guideline methodology training, establishment of dedicated guideline units within professional societies, and fostering international collaboration are essential (47, 48).
5. Mandate Patient and Family Engagement in Guideline Development: Our AGREE II assessment identified inadequate stakeholder involvement, with 78.79% of documents failing to consider patient/public perspectives. Incorporating the lived experiences of individuals with CHD is now an international expectation, aligned with WHO Handbook standards. Future guidance documents should explicitly describe how patients and family caregivers are engaged (e.g., as panel members, via surveys/focus groups) and how their input shapes final recommendations.
6. To further strengthen future CHD guidance documents, we also advocate for prospective guideline registration.In addition to improving methodological rigour and reporting quality, prospective registration of guideline protocols is a crucial step to enhance credibility. The PREPARE platform (https://www.guidelines-registry.cn)—the world's first prospective registry for clinical practice guidelines and expert consensus—supports bilingual (Chinese/English) registration and has registered over 5,000 documents since 2014. While AGREE II and RIGHT assess what has been reported, prospective registration ensures that the development process is planned transparently from the outset, reducing retrospective changes or selective reporting. We strongly recommend that future CHD guideline developers, particularly in China, register their protocols on PREPARE before initiating development—a practice increasingly adopted by major organisations and journals (49, 50).
7. High-quality CHD guidelines must align with the WHO Handbook. We recommend six WHO-endorsed practices: (1) PREPARE registration, (2) GRADE methodology, (3) stakeholder engagement including patients, (4) COI disclosure/management, (5) applicability planning (barriers, resources), and (6) transparent reporting (9). Chinese professional societies should establish national guideline units modeled on WHO’s GRC and collaborate with WHO Collaborating Centres. These steps will ensure CHD recommendations are scientifically valid, practical, and trustworthy.

Transforming CHD guidelines from expert compilations into scientifically robust, clinically actionable tools requires concerted efforts from developers, professional societies, journals, and funding agencies. By addressing these methodological and reporting deficiencies, we can enhance the translation of best evidence into optimal clinical practice, ultimately improving outcomes for the global CHD population.

This study also has several limitations. Firstly, only Chinese and English language documents were included, potentially excluding relevant guidelines in other languages. Secondly, while AGREE II and RIGHT are validated tools, certain domain assessments may involve subjective interpretation despite our measures to ensure consistency. Thirdly, the search was limited to November 2025 and was not updated during the revision process. Given the short time interval and the stable nature of methodological quality appraisal, the inclusion of newly published guidelines is unlikely to alter the overall conclusions. Nevertheless, future updates of this review may be warranted as the field evolves. Finally, we focused on methodological and reporting quality rather than clinical content validity, which represents a different but important aspect of guideline evaluation.

## 5 Conclusion

This systematic appraisal demonstrates that current CHD clinical guidelines and consensus statements exhibit significant quality deficiencies, particularly in methodological rigour, practical applicability, and reporting transparency. While international guidelines generally meet higher standards, Chinese documents show substantial room for improvement. The transition toward high-quality, evidence-based CHD guidelines requires systematic adoption of international development standards, strengthened implementation frameworks, transparent reporting, and ongoing update mechanisms. These improvements are crucial for ensuring that clinical recommendations are scientifically valid, practically feasible, and capable of improving patient care and outcomes in CHD management.

## Data Availability

The datasets presented in this study can be found in online repositories. The names of the repository/repositories and accession number(s) can be found in the article/Supplementary Material.
