# Supplementary material for: Appraisal of congenital heart disease guidelines and consensus
Source: Front Pediatr. 2026 Jul 24;14:1838468. doi: 10.3389/fped.2026.1838468 (PMC13447304; doi:10.3389/fped.2026.1838468)
Supplement: Supplementary file 1 [file Supplementaryfile1.pdf]

Supplementary File S1: Complete Search Strategies for All Databases

1. PubMed (U.S. National Library of Medicine)
Search Date: November 2025
Database Coverage: Inception to November 2025

Search Strategy:
("Congenital Heart Disease"[Mesh] OR "Heart Defects, Congenital"[Mesh] OR "congenital heart disease"[tiab] OR "congenital heart defect*"[tiab] OR "congenital cardiac malformation*"[tiab] OR "CHD"[tiab])
AND
("guideline"[pt] OR "guideline"[tiab] OR "practice guideline"[pt] OR "consensus"[tiab] OR "expert consensus"[tiab] OR "recommendation"[tiab] OR "statement"[tiab] OR "clinical practice guideline"[tiab])
Limits: English or Chinese language; Humans

2. Embase (Elsevier)
Search Date: November 2025
Database Coverage: Inception to November 2025

Search Strategy:
('congenital heart disease'/exp OR 'congenital heart defect'/exp OR 'congenital heart disease':ab,ti OR 'congenital heart defect*':ab,ti OR 'CHD':ab,ti)
AND
('clinical guideline'/exp OR 'practice guideline'/exp OR 'consensus'/exp OR 'guideline':ab,ti OR 'consensus':ab,ti OR 'expert consensus':ab,ti OR 'recommendation':ab,ti OR 'statement':ab,ti)
Limits: English or Chinese language; Humans

3. Web of Science (Clarivate Analytics)
Search Date: November 2025
Database Coverage: Inception to November 2025

Search Strategy:
TS=("congenital heart disease" OR "congenital heart defect*" OR "CHD" OR "cardiac malformation*")
AND
TS=("guideline" OR "practice guideline" OR "consensus" OR "expert consensus" OR "recommendation" OR "statement")
Limits: English or Chinese language; Articles, Reviews, Proceedings papers

4. CBM (China Biology Medicine disc, SinoMed)
Search Date: November 2025
Database Coverage: Inception to November 2025

Search Strategy:
(先天性心脏病 OR 先天性心脏缺陷 OR 心脏畸形 OR CHD)
AND
(指南 OR 共识 OR 实践指南 OR 专家共识 OR 推荐意见 OR 管理规范)
Limits: Humans

5. WanFang Data (万方数据)
Search Date: November 2025
Database Coverage: Inception to November 2025

Search Strategy:
主题:(先天性心脏病 OR 先天性心脏缺陷 OR 心脏畸形 OR CHD)
AND
主题:(指南 OR 共识 OR 实践指南 OR 专家共识 OR 推荐意见 OR 管理规范)
Limits: 中文学术期刊、学位论文、会议论文

6. CNKI (China National Knowledge Infrastructure, 中国知网)
Search Date: November 2025
Database Coverage: Inception to November 2025

Search Strategy:
(SU='先天性心脏病' OR SU='先天性心脏缺陷' OR SU='心脏畸形' OR SU='CHD')
AND
(SU='指南' OR SU='共识' OR SU='实践指南' OR SU='专家共识' OR SU='推荐意见' OR SU='管理规范')
Limits: 期刊、学位论文、会议论文

7. GIN (Guidelines International Network)
Search Date: November 2025
Database Coverage: Inception to November 2025

Search Strategy:

Search field: Guideline title or keyword

Search terms: "congenital heart disease" OR "CHD" OR "heart defect"

8. NICE (National Institute for Health and Care Excellence)
Search Date: November 2025
Database Coverage: Inception to November 2025

Search Strategy:

Search field: All NICE guidance

Search terms: "congenital heart disease" OR "CHD"

9. AHA/ACC Official Websites (American Heart Association / American College of Cardiology)
Search Date: November 2025
Database Coverage: Inception to November 2025

Search Strategy:

Sites: professional.heart.org, acc.org

Search terms: "congenital heart disease" OR "CHD" OR "congenital heart defect"

Filter: Guidelines & Statements

10. Yimaitong Guideline Channel (医脉通指南频道)
Search Date: November 2025
Database Coverage: Inception to November 2025

Search Strategy:

Search field: 指南标题 or 关键词

Search terms: "先天性心脏病" OR "CHD" OR "先心病"

Filter: 中文指南、专家共识

Supplementary Search
Hand-searching: Reference lists of all included documents were manually screened for additional eligible guidelines or consensus statements.

Note: All searches were conducted from database inception to November 2025. Only documents published in Chinese or English were considered. Search strategies were adapted as necessary for each platform while maintaining the core concepts: congenital heart disease AND (guideline OR consensus).
